# A case of nail-patella syndrome associated with thyrotoxicosis

**Published:** 2012-03-05

**Authors:** B Haras, F Vulpoi, Gh Onose

**Affiliations:** “Gr. T. Popa” University of Medicine and Pharmacy, Iasi; The Clinic Division of NeuroMuscular Rehabilitation of “Bagdasar-Arseni” Teaching Emergency Hospital, Bucharest, Romania

**Keywords:** hereditary onycho-osteodystrophy, nail-patella syndrome, osteoporosis, thyrotoxicosis

## Abstract

Nail-patella syndrome, also known as hereditary onycho-osteodystrophy, is a rare autosomal dominant disorder with pleiotropic phenotypic expression.

The present report is of a nail-patella syndrome patient, a 26-year-old female, admitted to our NeuroMuscular Rehabilitation Clinic Division for neurological symptoms, secondary to a severe spondylolysthesis with bilateral L5 pedicle fracture. During hospitalization, she was also diagnosed with mild thyrotoxicosis, but interestingly enough, the bone mineral density, assessed at multiple sites, was quasi-normal.

**Abbreviations** BMD – bone mineral density, BMI – body mass index, DXA – dual-energy X-ray absorptiometry, FT4 – serum free thyroxin, HOOD – Hereditary onycho-osteodystrophy, NPS – Nail-patella syndrome, TSH – thyroid-stimulating hormone

## Introduction

Nail-patella syndrome (NPS) also known as Fong disease, hereditary onycho-osteodystrophy (HOOD), Österreicher-Turner syndrome, or Turner-Kieser syndrome is a rare autosomal dominant condition, caused by anomalies of the LMX1B gene. Its incidence has been roughly estimated at 1: 50000 [**1,2**].

NPS is defined by three major features:

• Nail anomalies – in 80-90% of patients 3; they are bilateral and symmetrical; nails can be absent, hypoplastic, or dystrophic (discoloration, triangular lunulae, splitting, ridging, thinning) [**4,2**]

• Skeletal anomalies – present in virtually all patients; the most frequent are: absent or hypoplastic patellae, iliac horns, dysplastic elbows [**2,3,4**]

• Renal disease – found in approximately 40% of the NPS patients [**5**]: proteinuria, microscopic hematuria, hypertension; progression to renal failure has been reported in 5-14% of NPS patients [**2,3,5**]. 

There is a great inter- and intrafamilial variability of the NPS clinical manifestations and literature suggests it is common for the disorder to go undiagnosed for several generations in affected families [**2**].

Iliac horns, observed in up to 70% of the patients [**1**], are the patognomonic radiological features of the disorder. They are asymptomatic, symmetrical findings, consisting in bone spurs arising from the iliac crests, sometimes detectable on physical examination.

The patellae may be absent or hypoplastic; fragmentation is frequent, resulting in lateral slippage during knee flexion [**4,6**]. Radial heads are often hypoplastic, causing subluxations of the elbow. Complications such as joint effusions and secondary arthrosis of the affected joints may cause pain and functional limitations. A wide range of other skeletal anomalies can be found (with lower frequency) in NPS patients: genu recurvatum, varum or valgum, hypoplasia of cruciate ligaments, pectus excavatum, scoliosis, increased lumbar lordosis, spondylolisthesis, spondylolysis, spina bifida, etc. [**2**]

Other features described in NPS patients are: glaucoma [**1**], gastrointestinal symptoms (constipation, irritable bowel syndrome), lean habitus and difficulty to gain weight, depression, attention deficit disorder [**2,7**].

Though NPS is associated with multiple skeletal anomalies and often low body mass index (BMI), it has not been proved to associate with severe decreases of bone mineral density (BMD). One report described a case of NPS with osteoporosis [**8**]. A clinical study found that NPS adult patients had a bone mineral density (BMD) lower than normal adults, but still, in the normal to osteopenic range; their results also showed a significantly higher prevalence of fractures among the NPS patients, generally occurring before puberty [**9**]. This may suggest that bone fragility in these patients may be due to other causes, such as altered quality of the matrix, or abnormal turnover [**10**].

Thyrotoxicosis has never been described as a feature of NPS and it was probably an incidental, non-related finding in our patient. In this particular case, given her medical history of fractures, without a clear account of a previous significant trauma, the presence of thyrotoxicosis, a known risk factor for osteoporosis [**11,12**] , led us to further investigate the skeletal integrity.

## Case Report

A 26-year-old female patient, previously diagnosed with NPS, was admitted to our clinic for neurologicall rehabilitation. She presented cauda equina syndrome, secondary to severe anterior spondylolysthesis of L5 on S1, which had been operated 3 times, with partial amendment of the neurological deficits. 

According to the patient, she had first developed severe low back pain in 2002, when she was diagnosed with anterior spondylolysthesis L5-S1 and bilateral fracture of the L5 pedicles; she was first operated in 2003 (posterior spondylodesis with autologous bone graft and fixation with a plate and screw system); the neurological symptoms first occurred in 2005 (when the osteosynthesis material was extracted). In 2009, she experienced a worsening of the motor impairment, with the onset of urinary incontinence and underwent spinal surgery again (anterior spondylodesis with rib bone graft and fixation with a dual rod system). The existence of a traumatic precipitating factor, as well as the exact progression of her symptoms remained unclear.

The chief complaints at admission were: paraplegia with L3 neurological level and moderate motor deficit, affecting predominantly the left lower limb, paresthesia in the left lower limb and urinary incontinence. 

Her medical history revealed:

• Multiple drug allergies (nonsteroidal anti-inflammatory drugs, metamizole, mepivacaine)

• Allergic asthma

• Hypoplasia of the sternal manubrium (diagnosed in 2010, by computed tomography)

• Dislocatable left hip – operated in 2010

• Dislocatable left patella – operated twice, in 2003 and 2007

• Severe chronic constipation

**Fig. 1:**
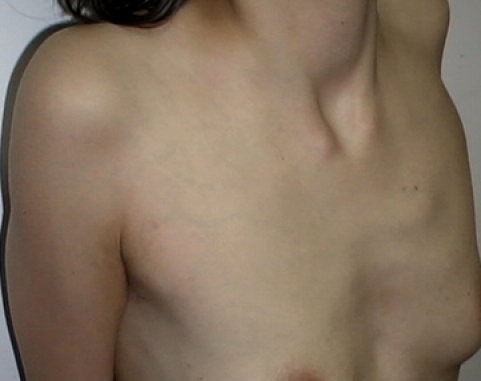
Pectus excavatum

**Fig. 2:**
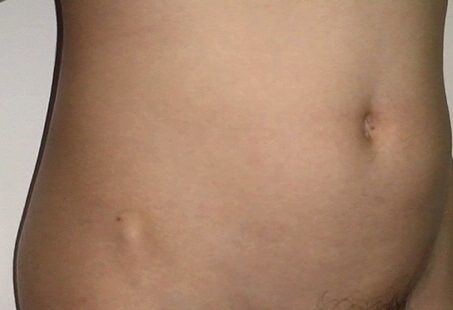
Visible anterior iliac horn

Aside from the chief complaint, physical examination found multiple features associated with NPS, especially skeletal anomalies:

• Asthenic habitus, BMI of 15,807 kg/m² (with normal dietary intake)

• Global, symmetrical muscle hypotrophy 

• Global joint hypermobility

• Pectus excavatum (**Figure 1**)

• Increased lumbar lordosis

• Bilateral iliac horns (**Figure 2**)

• Moderate left knee instability

The patient’s nails, surprisingly, showed no abnormalities. 

Ophthalmologic examination found bilateral hyperopic moderate astigmatism, but no glaucoma. Laboratory results were normal, except for a urinary tract infection, probably due to urinary incontinence and bladder catheterization. Repeated tests found no proteinuria.

In addition, the physical examination found that the patient had warm, moist skin and a discrete tremor of the hands. This prompted us to assess the thyroid function. The results showed a suppressed thyroid-stimulating hormone: TSH 0,252 μIU/ml (normal 0,4-4,70 μIU/ml), a high level of serum free thyroxin: FT4 26,3 pmol/l (normal 10-23 pmol/l) and elevated thyroid antibodies: thyroid peroxidase antibodies 368,14 IU/ml (normal 0-5,61 IU/ml); thyroglobulin antibodies 31,12 IU/ml (normal 0-4,11 IU/ml), leading to the diagnosis of thyrotoxicosis, probably of autoimmune etiology.

Given the history of previous fractures, prolonged pain and motor impairment, resulting in mobility limitations, associated with the presence of thyrotoxicosis, we decided to further explore the bone mineral density of the patient. When informed about this test, she produced the results to a BMD DXA (dual-energy X-ray absorptiometry) investigation she had undertaken 3 months before. As seen in **Table 1**, the T score values ranged from normal to mild osteopenia for all regions explored.

**Table 1:** BMD – DXA assessment: detailed T score values. We did not consider the L4 vertebra a valid region for this test,since it had born a metal screw

Region	T score (young adult)	Z score (age-matched)
Left femoral neck	-0,6	-0,1
Right femoral neck	0,4	0,9
Left Wards triangle	-0,2	0,2
Right Wards triangle	0,0	0,4
Left trochanter	-1,1	-0,5
Right trochanter	-0,8	-0,2
Left total hip	-0,8	-0,2
Right total hip	-0,2	0,3
L1	-1,2	0,4
L2	-1,4	0,6
L3	0,6	1,3
L1-L2	-1,3	-0,5
L1-L3	-0,5	0,3

## Discussion

The gene implicated in NPS, LMX1B (LIM homeobox transcription factor 1-beta), is located on the long arm of chromosome 9 (9q34) [**2,9**]. It has been demonstrated that this transcription factor plays an important role in skeleton, kidney and eye development [**2,13**], but the full extent of LMX1B’s regulatory functions is still unclear. To date, more than 100 loss-of-function mutations of this gene have been identified in NPS patients [**9,14,15**].

Our patient was diagnosed with NPS in 2010. Her DNA sequence analysis showed a heterozygous missense mutation in exon 5 of LMX1B gene: c.713G>A (p.Arg238His), which had never been described before (GenBank Accession no NM_002316.2). Her mother was clinically diagnosed with NPS, having nail aplasia and a dysplastic patella, but no other manifestations. The lack of phonotypical similarity between daughter and mother is not surprising, given the important individual variability of NPS clinical features.

Aside from the unusually severe, invalidating spondylolysthesis, accompanied by numerous other skeletal anomalies, the case was interesting because of the association with thyrotoxicosis and almost normal BMD. To date there is no evidence of this gene playing a role in the development of the hypothalamic pituitary thyroid axis, which is why we assumed this association was incidental.

We initially inferred that the presence of osteoporosis was highly probable in this patient for several reasons:

• NPS, a disorder affecting the limb development may interfere with bone mineralization

• Extensive anamnesis did not reveal a clear traumatic cause for the pedicle fractures

• The patient had a history of prolonged (about 8 years) severe pain, motor impairment, repeated surgical interventions, that had significantly limited her physical activities

• The patient also had thyrotoxicosis with an undetermined duration; adult thyrotoxic patients generally have an increased bone turnover [**11**], with a tendency to bone loss, probably due to accelerated resorption, not compensated by a similar increase in bone formation [**12**]

• She had a significantly low BMI, which according to her account was not due to a recent loss of weight

BMD assessment by DXA contradicted this hypothesis. We only found values of the T score consistent with mild osteopenia in the lumbar spine (L1-L2). Interesting findings were the lower values of the T score in the left hip, in comparison to the right one; this correlated with the severity of the motor impairment, which was more pronounced in the left lower limb. Therefore, it seems logical to assume that the (mild) decrease in BMD seen in this patient had the limitation of physical activity as main etiological mechanism, and not thyrotoxicosis or the underlying genetic condition. 

Since the patient was young, with no sign of renal involvement or cardio-respiratory dysfunction, our objectives were to improve her neurolocomotor performance and enhance her autonomy. We fitted her with a textile brace for the left knee, a left ankle-foot orthosis and a contralateral Canadian crutch. Since her spine was surgically stabilized, she has undergone a complete kynesitherapy program. We started her on intermittent catheterization, to alleviate the need for adult diapers, and 4-5 catheterizations a day proved sufficient to manage the urinary incontinence. She only experienced moderate pain after prolonged walking or standing and analgesia was satisfactorily achieved with acetaminophen. 

Upon discharge, she was able to walk (for distances longer than 500 m) and climb stairs. We prescribed vitamin D, calcium, B complex supplements, neutrophics, acetaminophen, and we referred her to an endocrinology service for further assessment and treatment.
